# Origins of 1/f noise in human music performance from short-range autocorrelations related to rhythmic structures

**DOI:** 10.1371/journal.pone.0216088

**Published:** 2019-05-06

**Authors:** Ian D. Colley, Roger T. Dean

**Affiliations:** The MARCS Institute for Brain, Behaviour and Development, Western Sydney University, Sydney, NSW, Australia; University of British Columbia, CANADA

## Abstract

1/*f* fluctuations have been described in numerous physical and biological processes. This noise structure describes an inverse relationship between the intensity and frequency of events in a time series (for example reflected in power spectra), and is believed to indicate long-range dependence, whereby events at one time point influence events many observations later. 1/*f* has been identified in rhythmic behaviors, such as music, and is typically attributed to long-range correlations. However *short*-range dependence in musical performance is a well-established finding and past research has suggested that 1/*f* can arise from multiple continuing short-range processes. We tested this possibility using simulations and time-series modeling, complemented by traditional analyses using power spectra and detrended fluctuation analysis (as often adopted more recently). Our results show that 1/*f*-type fluctuations in musical contexts may be explained by short-range models involving multiple time lags, and the temporal ranges in which rhythmic hierarchies are expressed are apt to create these fluctuations through such short-range autocorrelations. We also analyzed gait, heartbeat, and resting-state EEG data, demonstrating the coexistence of multiple short-range processes and 1/*f* fluctuation in a variety of phenomena. This suggests that 1/f fluctuation might not indicate long-range correlations, and points to its likely origins in musical rhythm and related structures.

## Introduction

1/*f-*type correlations have been identified in numerous physical and biological systems, often being described as ‘ubiquitous’ [1, 2]. This phenomenon refers to a pattern of noise over time that exhibits a roughly 1:-1 relationship between power and frequency in a log-log plot of a time series that has been converted to the frequency domain [3]. 1/*f* can also be thought of as a 1:1 relationship between the amount of fluctuation within a window of observations, and the size of the window as the window is incremented [4]. 1/*f* is often conceptualized as the center of a continuum of noise color that includes white noise or random fluctuations at one end, and red noise (also called Brownian motion) or deterministic fluctuations at the other end. 1/*f*, or pink noise, therefore represents a flexible system that fluctuates within a set of constraints [5]. Systems exhibiting 1/*f*-type noise are stable but adaptable, and thus have been suggested as indicative of a healthily functioning biological system [4, 6–14]. However, their widespread occurrence both in physical and biological systems somewhat undermines this proposed interpretation, suggesting rather that 1/f is simply compatible with normal function.

As indicated, the type of noise color is usually quantified using a scaling exponent, which represents the slope relating log-power to log-frequency in a power-spectral density (PSD) plot. White noise has a slope of 0, red or Brownian noise has a slope of -2, and pink or 1/*f* noise has an intermediate slope of -1. These slopes are typically represented as absolute values by the scaling exponent β. As such, β values between 0 and 2 are considered indicative of a 1/*f*-type process. We note that such simple log-power characterizations are by no means definitive fulfilments of the mathematically more stringent requirements for identifying a true 1/f process [1, 15], which we will not elaborate here: an in depth book on LRD time series analysis is available [16]. Our concern is with the origins of 1/f in musical performance, and for this purpose the simpler broader circumscription of 1/f is appropriate. Beran also reveals some of the complexities of 1/f behavior in pianists and composers in a book focused on musicology [17]; cf pp 158–160), and has more recently discussed the application of statistical and algebraic ideas to music composition [18].

Studies of human behavior more broadly have shown 1/f fluctuation in tasks such as grip force [19], self-paced tapping/time estimation [20–22], simultaneous force and time estimation [23], stride interval [24], synchronization tapping [25, 26], word naming [27], reaction times [28], other cognitive processes [3, 29], and also in brain activity during image observation [30]. 1/*f* fluctuations are also seen in routine stable standing balance [31, 32], suggesting that this pattern of noise may be a fundamental aspect of the human cognitive and motor system, and compatible with healthy behavior.

Given that many of these 1/f systems involve repeated time estimation, the presence of 1/*f*-type fluctuations has been identified in many complex behaviors as well, especially musical contexts, since music is a quintessentially temporal art. This was originally done by recording a radio broadcast for 12 hours and identifying 1/*f* in the amplitude of the signal [33]. Although this particular study was challenged [34], the spectral properties of music it reported have become widely accepted. More recently, postural sway in trombonists has been shown to exhibit 1/*f* [35], as has pulse-interval timing in piano performance [36, 37], and synchronization tapping over very long sequences [38]. Perceptually, music listeners tend to prefer “humanized” rhythms with 1/*f*-like noise added to remove perfect regularity [38], but ‘humanizing’ is also practiced in music production by other statistical means. There have been numerous other examples of 1/*f* in music since the 1970’s [39–44]. One compelling case of 1/*f* in music performance is an analysis of a performance by a professional drummer [45]. In this paper, the researchers found 1/*f* correlations in both the timing and amplitude of a hi-hat recording.

They, along with many other research teams [32, 36, 37, 46–49], attribute this and similar findings mechanistically to *long-range dependence* (LRD; often also termed long-range correlations) in the temporal structure of human behavior, meaning events at one time point have influence on events dozens, or even hundreds of observations later. However, consistent with a wide range of other literature concerning 1/f in physical, internet [50], and biological processes [1, 15] we show here that this may not be an appropriate interpretation of 1/*f-*type fluctuations in music performance, or in other processes.

Putting aside in depth discussion of what constitutes stringent LRD, we note that it has been known since at least 1980 that LRD can arise from the continuing co-existence of multiple short range processes (short range dependence, SRD), even for example from multiple 1^st^ order autoregressive components of different scale [51]. In a more recent discussion of mechanisms for generation of 1/f noise [15] such superposition of short range processes, as well as instabilities in attention are considered as possible alternates, leading to the suggestion that it is the kinetic nature of the short-range processes (whether attentional fluctuations or otherwise) that are important in determining whether 1/f-type noise emerges.

Returning to music, in early preliminary work, one researcher made the case that 1/*f* could indeed arise from certain short-range, autoregressive processes (SRD) when they occur over multiple time lags [19]. In this interesting report, a series of simulations showed that by applying moving average filters of varying window sizes—where the window sizes represent different time lags—to a white noise signal, the resulting series will show very clear 1/*f* properties when analyzed using PSD plots. Importantly, these two conditions (short range processes and multiple time lags) are analogous to most genres of music performance, in which short range corrective processes operating repeatedly over multiple constant time lags are a well-established finding [52–56], and movement timing is structured hierarchically into timescales (e.g. beat or pulse sequences become bars, which become phrases; [57]. This prompted us to extend work on short-range 1/*f* simulations [19] and apply the concept to observed music performance by re-analyzing in-depth the aforementioned hi-hat drumming data [45], and running a series of short-range auto-regressive moving average (ARMA) simulations. We show that a parsimonious, SRD interpretation of 1/*f* in human rhythmic performance—which is consistent with the short-range models of movement timing in the sensorimotor synchronization literature [54–56, 58]—may be preferable to the more nebulous LRD interpretation. We consider this issue further in the Discussion and Conclusions section. For a more thorough tutorial on analytical differences between SRD and LRD, see work by Stadnitski [59].

**Fig 1:**
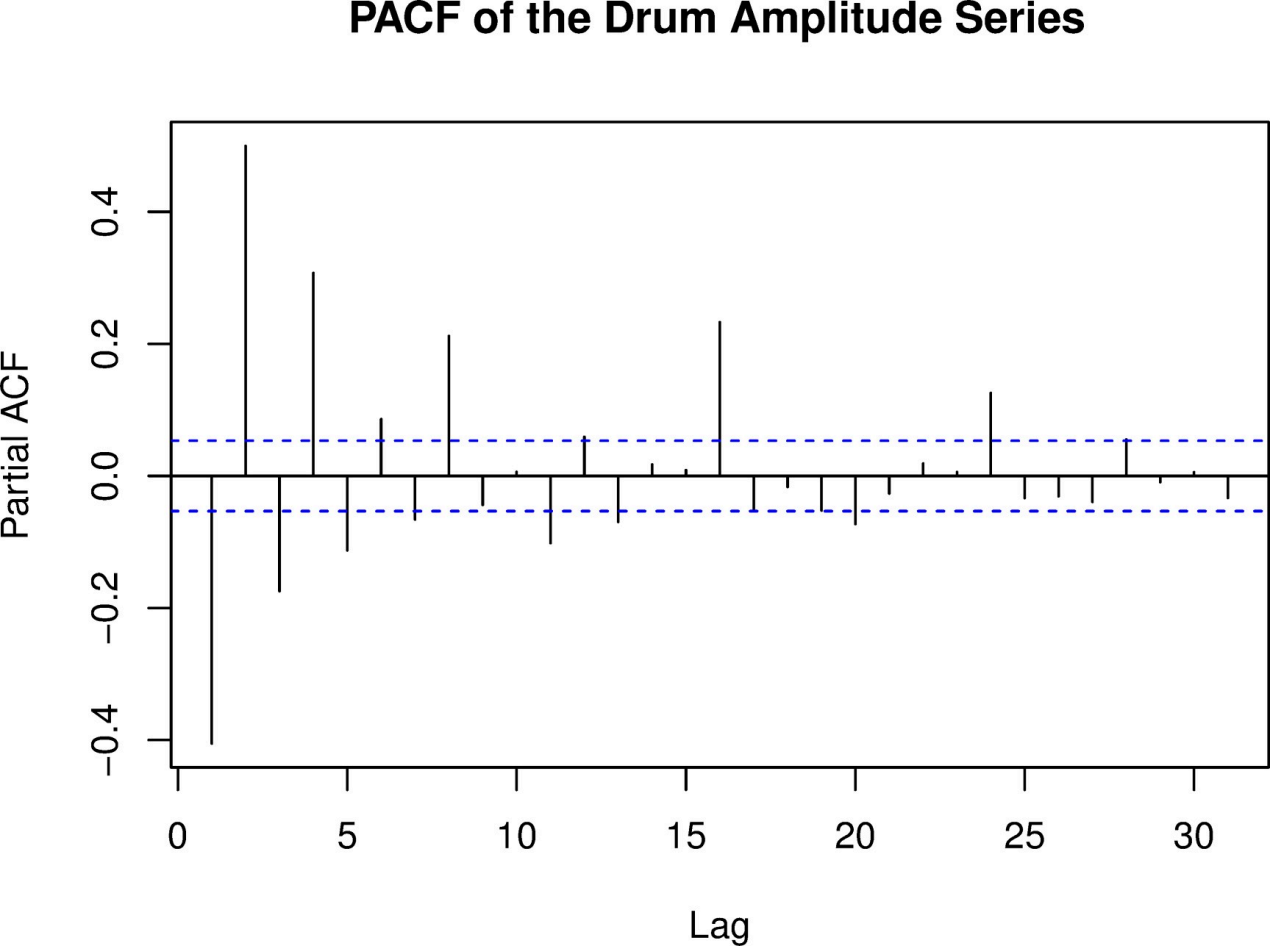
The partial autocorrelation function of the amplitude measurements of the hi-hat drum series. Values outside the dotted lines are significant. The non-sequential significant lags up to lag-24 show that drummer is structuring the force of his drum hits according to metrically relevant events.

Our initial assessment of the hi-hat drumming dataset suggested that the time series is indeed operating over multiple short-range time lags, which again, makes sense intuitively given the different levels of metrical structure that underlie musical rhythms. This is evidenced in the partial autocorrelation function of the amplitudes of the data set, which shows significant lag coefficients at orders 1–8, then 16 and 24 (**Fig 1**), suggesting a strong influence up to and slightly beyond the timescale of the musical bar (which is equivalent to sixteen 16^th^ notes/semiquavers, or 16 lags), in addition to the obvious importance of lag-1 (the previous pulse). Thus, we considered further whether the 1/*f* correlations identified in this recording and other types of rhythmic performance may not actually reflect long-range processes, but instead arise from relatively short-range correlated autoregressive processes. From here on, we will focus our analysis on temporal intervals (inter-pulse intervals of the drum hits, specifically), rather than amplitudes, as this pertains more directly to our interest—as well as the interests of other research teams—in correlational structures in musical timing.

Although addressing this specific concern is the primary motivation for our study, our use of time series simulations is also exploratory as we hope to identify a variety of short-range structures that can lead to 1/*f*-like noise. To do this, we used two measures of time series noise: PSD plots, and the more modern detrended fluctuation analysis (DFA). DFA has become very popular for fractal time series analysis [4, 7–9, 13, 59, 60] and so we wanted to test if it is sensitive to the same short-range conditions as PSD.

Our specific questions are addressed as follows: 1) Will ARMA (autoregressive moving average) simulations of the recorded hi-hat time series produce 1/*f* correlations? If so, 1/*f* in this context may not reflect long-range processes, but instead be a product of autoregressive processes operating over multiple time lags and over a short-range of influence. 2) Can apparent long-range structure, detectable in both spectral (PSD) and fractal (DFA) analysis, result from a fairly wide range of auto-regressive (AR) structures? Specifically, can AR structures in which the involved lags are separated by several gaps, and in which the coefficients differ significantly lead to 1/*f*-like noise? This could readily arise in music because of its hierarchical structure, such that the occurrence of beats (also called pulses) are grouped into sequences of 1:2:4:8, or 1:3:6:9, or less commonly 1:5:10:15. 3). Will 1/*f-*type noise in a range of non-musical human activities also co-exist with short-range features that might again be explanatory? To test this, we analyzed several publicly available datasets (from physionet.org and elsewhere) of other time series where 1/*f* noise might be found, specifically heart beat intervals, stride intervals, and a resting state EEG recording.

Understanding our simulations and modeling procedures is crucial for understanding the results. Thus in what follows we describe the methods for each research question alongside the results, with a general Methods section following at the end.

## Results

Before investigating the hi-hat data and running related simulations, we wanted to compare PSD and DFA to see if they would yield consistent results on a dataset known to produce 1/*f* noise. To do this, we replicated past 1/*f* simulations [19] and analyzed the series using both PSD and DFA. The simulation involved three steps. First we generated a white noise series, *WN*, of length 1,024 and with arbitrary time units. Second, we made three copies of the white noise series, each with a different moving average, *MA*, filter applied. The *MA* filters differed in their window size, *q*. In other words, *q* is the number of successive events in the *WN* series that were averaged by the *MA* filter. Third, we summed all four series with different weights, *θ*, given to the each series, where *θ* represents the influence of each timescale/*MA* window size. As a formula, the process is:

$$WN\text{+}\theta_{1}MA\text{(}q_{1}\text{)+}\theta_{2}MA\text{(}q_{2}\text{)+}\theta_{3}MA\text{(}q_{3}\text{)}$$

In summary, the simulation starts with an uncorrelated process (white noise), and then adds repetitive influence at different lags (the moving average filters). We expanded on this by testing a variety of simulations in which we adjusted *q* and *θ* according to the following criteria: 1) all *θ* weights the same (simulation number 1) to simulate equal influence from multiple window sizes/temporal processes; 2) window sizes of small values representing a small range of influence with θ weights increasing with window size; 3) window sizes increasing exponentially to produce a wider range of influence and θ weights increasing with window size; 4) the same window sizes as points 2 and 3, but with large θ weights paired with small window sizes, in order to test an unusual condition where a process with more frequent influence (i.e. a shorter timescale) produces more extreme events; normally, heavily weighted events are less frequent in LRD signals. In all cases, we chose to use small integer *θ* weights for each *MA* series to test whether a 1/*f-*type shape would emerge from relatively weak influence from each simulated timescale.

**Fig 2:**
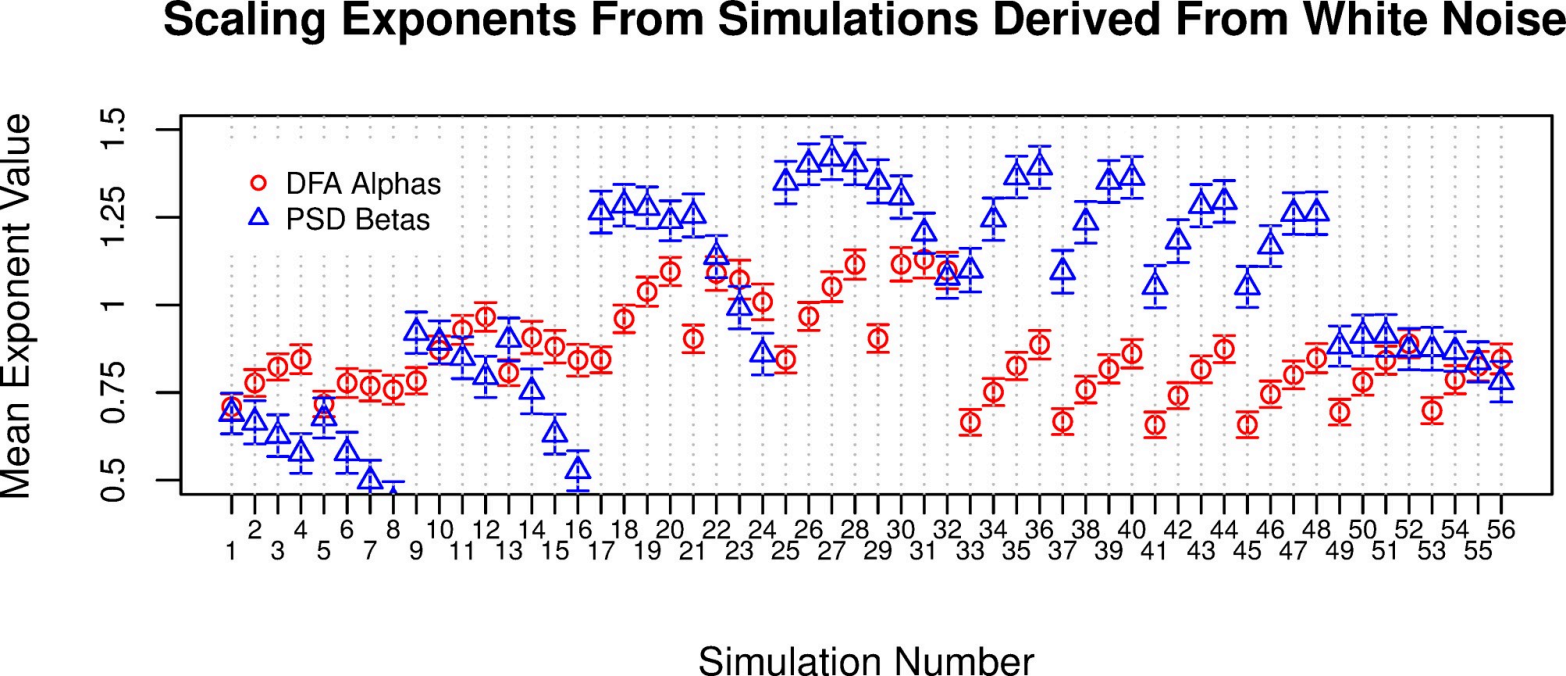
Alpha and beta values from simulations derived from white noise. There were 1,000 iterations of each simulation, each containing 1,024 observations of arbitrary time units. Each simulation iteration was analyzed with PSD and DFA, with the corresponding mean exponents plotted on the *y*-axis. Error bars are standard deviation. S1 Table in the supporting information lists the specific formulae used for each simulation.

The simulations (56 in total) were iterated 1,000 times each, and all iterations were analyzed using PSD and DFA. Given the large number of simulations, we have listed all formulae alongside the corresponding mean and standard deviation for DFA α and PSD β in S1 Table in the supporting information. A summary of these simulations is depicted in **Fig 2**, which shows the mean DFA α and mean PSD β values for all 1,000 iterations of each simulation. While some of the simulations were closer to white noise or Brownian noise, many of the simulations showed 1/*f*-like shape, even though any serial influence was relatively short-range.

For the hi-hat data, we first fit the series to ARMA and ARIMAX models using the R function auto.arima from the ‘forecast’ package, in order to determine the autoregressive components of the drumming performance. Here the covariate *X* is an essentially isochronic representation of the drummer’s realized rhythmic structure. Rests, being periods of inactivity, were included so that the complete data series was used unchanged. In other words we looked at the onset times of drum hits regardless of intentional silences (“rests”), and the whole autocorrelation context was thus considered. The *ARIMA* part of the models represents the autoregressive inter-pulse interval error correction of the drum hits. The models showed extremely good fit (RMSE < 0.01, in relation to a mean inter-pulse interval of c. 156 ms), and (4, 0, 1) or (5, 0, 0) structures were amongst the optimal models, as judged also by the Bayesian Information Criterion (which penalizes model complexity). Here the (*p*, *d*, *q*) description defines the number of autoregressive (AR) lags, the degree of differencing (I), and the number of moving average (MA) lags. For simplicity of interpretation and subsequent simulation, we forced the model to exclude differencing, so they were effectively ARMA and ARMAX models.

**Fig 3:**
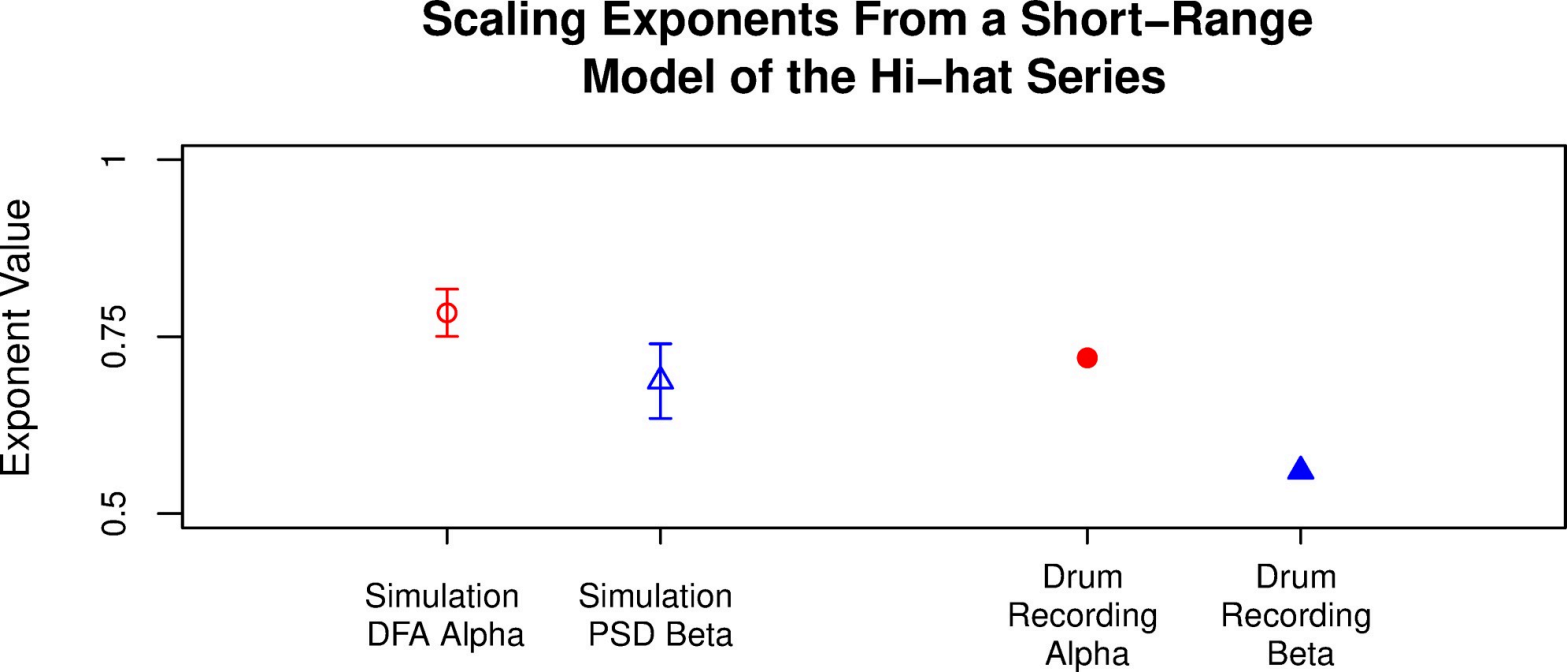
Scaling exponents from short-range ARMA simulations. Each simulation used 1,024 observations and was analyzed using DFA and PSD, with mean scaling exponents plotted along the *y*-axis. Error bars are standard deviation. For comparison, we also plotted the DFA and PSD results of the human drum performance from which the simulations were derived. The ARMA structure was AR(-.5, .48, .19, .03) MA(.93).

We pursued the ARIMA(4,01) structure, which was a slightly better model than ARIMA(5,0,0). Using the coefficients associated with these lags (**Fig 3**), and the distribution of the inter-pulse intervals from the drum data (which provided the error variance), we simulated the hi-hat series using the R function arima.sim. This allowed us to see if 1/*f*-type fluctuations—typically interpreted as LRD—would appear in a series that modeled highly expert human performance using short-range influence (just four lags). In theory this simulation should not show LRD since the largest significant lag is within a window of only four observations (or four metrical divisions in this case), which is a relatively short-range of influence. However, this structure actually produced moderately LRD 1/*f*-type fluctuations. Across the 100 iterations, the mean α was .78, and the mean β was .70 (see Fig 3).

**Fig 4:**
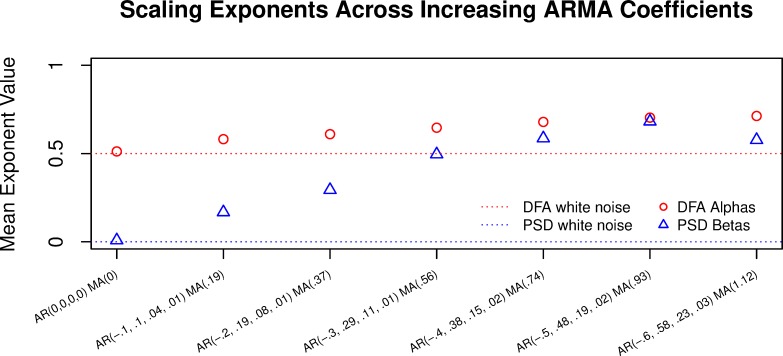
The horizontal dotted lines represent white noise values. Any of the alpha values above 0.5 and any beta values above 0 suggest a long-range correlated structure, given all the data are in the range (0–1]. The ARMA coefficients are listed along the *x*-axis for each simulation.

We also ran simulations using the same number of AR and MA lags (1–4 and 1 respectively), but with different coefficients. We preserved the general structure of the ARIMA model that had been fitted to the hi-hat series such that the AR1 coefficient was the largest AR magnitude and negative (auto-corrective), and the remaining coefficients were proportional to AR1 (e.g. AR2 was always of similar magnitude to AR1, but positive). Model 1 set all coefficients to zero, which is white noise (α = .5, β = 0). After that, AR1 was incremented by .1 for each model. Most of the simulations after model 1 were suggestive of 1/*f* when analyzed with PSD and DFA (see **Fig 4**).

Next, we tested whether 1/*f*-like structures would show up in ARMA models with non-significant AR lags in between significant lags. This is unusual for an ARIMA model, which typically has sequential lags of decreasing coefficients until a lag is not significant. However, the use of non-sequential lags is analogous to human rhythmic performance, wherein some beats fit into levels of a hierarchy of pulses, bars, and phrases, making intermediary pulses less important as already noted by the metrical influence shown in the amplitude time series of the hi-hat performance. Thus, influence from these metrically significant points in a rhythmic series could occasionally produce 1/*f*-like effects, despite the unusual ARMA structure. In these simulations, the largest lag used was 15, which presents an interesting mid-range of influence. Whereas our previous ARIMA simulations have only been 4^th^ order, and long-range correlations reflect influence from dozens or even hundreds of observations back, these gapped simulations are testing for LRD with a model order that goes just beyond a typical ARIMA range for rhythmic performance. The results of these simulations (100 iterations each) are listed in **Table 1**. We included a significant AR1 for all simulations, as this reflects influence from the immediately preceding event, which is expected in most performance. The next significant lag was of order 3, 4, or 5 depending on the musical meter being simulated (3/4, 4/4, or 5/4 respectively). These lags represent influence from a pulse/rhythmic event that is 3, 4, or 5 observations in the past. Any later significant lags represent influence from pulses further back in a rhythmic series. The MA1 component for all simulations was kept at .5.

**Table 1:** Scaling exponents from simulations lag gaps.

Simulated Meter	AR Structure	α	β
	-.5 0 0 .4	.57	.56
	.5 0 0 -.4	.42	1.12
	-.5 0 0 -.4	.40	-.07
	.5 0 0 .4	1.01	1.39
	-.4 0 0 .3 0 0 0 .2	.60	.17
4/4	.5 0 0 -.4 0 0 0 -.3	.45	1.08
	-.5 0 0 -.4 0 0 0 -.3	.35	-.15
	.4 0 0 .3 0 0 0 .2	1.07	1.32
	-.4 0 0 .3 0 0 0 .2 0 0 0 .1	.37	.08
	.4 0 0 -.3 0 0 0 -.2 0 0 0 -.1	.5	.98
	-.4 0 0 -.3 0 0 0 -.2 0 0 0 -.1	.33	-.13
	.4 0 0 .3 0 0 0.2 0 0 0 .1	1.11	1.36
	-.5 0 .4	.58	.19
	.5 0 -.4	.54	1.11
	-.5 0 -.4	.37	-.15
	.5 0 .4	1.15	1.56
	-.5 0 .4 0 0 .3	.56	.13
	.5 0 -.4 0 0 -.3	.42	1.00
3/4	-.5 0 -.4 0 0 -.3	.42	-.19
	.5 0 .4 0 0 .3	1.13	1.37
	-.4 0 .3 0 0 .2 0 0 .1	.65	.25
	.4 0 -.3 0 0 -.2 0 0 -.1	.44	.83
	-.4 0 -.3 0 0 -.2 0 0 -.1	.43	-.04
	.4 0 .3 0 0.2 0 0 .1	1.37	1.47
	-.5 0 0 0 .4	.56	-.07
	.5 0 0 0 -.4	.48	1.07
	-.5 0 0 0 -.4	.43	-.02
	.5 0 0 0 .4	1.09	1.34
	-.5 0 0 0 .4 0 0 0 0 0 .3	.55	.19
	.5 0 0 0 -.4 0 0 0 0 -.3	.48	.96
5/4	-.5 0 0 0 -.4 0 0 0 0 -.3	.40	-.07
	.5 0 0 0 .4 0 0 0 0 .3	1.00	1.24
	-.4 0 0 0 .3 0 0 0 0 .2 0 0 0 0 .1	.54	.25
	.4 0 0 0 -.3 0 0 0 0 -.2 0 0 0 0 -.1	.49	.98
	-.4 0 0 0 -.3 0 0 0 0 -.2 0 0 0 0 -.1	.42	.12
	.4 0 0 0 .3 0 0 0 0 .2 0 0 0 0 .1	1.13	1.36

These simulations include non-significant lags (represented by zeros) in order to represent the possible influence of different time scales in musical meters. We simulated three meters (4/4, 3/4, and 5/4) and adjusted the sign of the coefficients to see the combined effect of negative and positive correlations at different lags. The magnitude of coefficients had to be reduced for the larger AR structures in order to keep the simulations stable. MA1 was fixed at .5 for all simulations.

The main finding from these simulations is that the sign of the coefficients matters most in producing 1/*f*-like effects, and the number of intervening, non-significant lags (i.e. coefficient = 0) does not change much. Specifically, when all AR lags are positive, the resulting exponents from DFA and PSD show 1/*f*. This makes sense as it introduces small positive correlations at different timescales, meaning the series will drift similarly both at small scales (AR1) and larger scales (e.g. AR5, AR10, AR15), which is the characteristic shape of 1/*f*. Most simulations containing negative coefficients produced stationary series close to white noise, or anti-correlated (exponents less than white noise values) series. This also makes sense as negative lagged correlations represent corrective processes that keep the series bounded and prevent drift. One curious condition is when AR1 is positive, but all following significant lags are negative (3^rd^ row from the bottom of each meter box in Table 1). Here the DFA and PSD results diverged, such that PSD showed 1/*f*, but DFA showed white noise. These simulations are exploratory and ARIMA structures like these may not appear often in experimental tests, but it is worth noting that the two methods of analysis are not always consistent.

Next we ran similarly exploratory simulations using ARMA structures with just one or two lags to test if 1/*f*-type fluctuations might be observed in a time series with an extremely short range of influence. More specifically, we wanted to assess purely AR process descriptions to eliminate the possibility that the MA (error description) term is a critical feature. We expected to be able to show this since an MA model can always be represented instead as an AR one. As a preliminary, we noted that the hi-hat drumming series was well modeled as AR (5, 0, 0), and we confirmed that even simulating from AR (1, 0, 0) and (2, 0, 0) models generates 1/*f* providing the first AR coefficient is positive. This observation is consistent with an earlier simulation study [59]. We then considered the following criteria to test a further variety of short-range conditions: 1) only one or two coefficients were used, and these were associated with two AR lags, two MA lags, or one of each; 2) the signs of the coefficients were both positive, both negative, positive followed by negative, or negative followed by positive; 3) the magnitude of the coefficients was held at .5 for the first one, and .4 for the second one. We again ran 1,000 iterations of each simulation, and the results are listed in **Table 2**. Consistent with the previous round of simulations, conditions in which the coefficients are positive seem to produce scaling exponents that are suggestive of 1/*f*-type fluctuations.

**Table 2:** Scaling exponents from simulations with only two lag components.

ARMA Structure	Mean DFA α	Mean PSD β
AR2 = 0.5, 0.4	1.02	1.08
AR2 = 0.5, -0.4	.52	.48
AR2 = -0.5, 0.4	.34	-.56
AR2 = -0.5, -0.4	-.29	-.62
MA2 = 0.5, 0.4	.66	.69
MA2 = 0.5, -0.4	.49	.46
MA2 = -0.5, 0.4	.47	-.44
MA2 = -0.5, -0.4	.11	-1.02
AR1 = 0.5 MA1 = 0.4	.73	1.15
AR1 = 0.5 MA1 = -0.4	.57	.15
AR1 = -0.5 MA1 = 0.4	.50	-.13
AR1 = -0.5 MA1 = -.04	.21	-.62

These simulations were made with just two lags and different combinations of positive or negative coefficients. Even in these very short-range structures, the shape of a time series can show 1/f-type fluctuations if the coefficients involved are positive.

Finally, we wanted to consider whether the range of ARMA structures we have found so far that are capable of generating 1/*f* features, co-exist with 1/*f* in a small but diverse set of other human processes, and hence might possibly be explanatory in a wide range of contexts. This could be anticipated from the earlier mathematical and statistical work [1, 15]. Specifically, we analyzed movement (walking gait time intervals), physiology (heart beat intervals), and cognition (a resting EEG recording). We describe both the AR structure using AR models, and the apparent long-range structure using DFA and PSD. These summaries are listed in **Table 3**.

**Table 3:** Coexistence of 1/f and SRC in time series data for diverse human processes.

Process	AR features (coefficients)	1/f features	Data source (see Methods for details)	Comments
**Movement:**				
Unconstrained Gait	p = 9 (.30, .19, .10, .06, .04, .01, .06, .01, .07)	α = .91 β = .62	Physionet	DFA α strongly suggests 1/f; PSD shows moderate 1/f-type shape. The AR model includes a mean of 1.04 seconds.
Drumming	p = 5	α = .78 β = .70	Räsänen et al (2015)	Analyzed in the present paper. Such data together with tapping data are often considered ‘time estimation’.
**Physiology:**				
Control Heart Beat	p = 3 (1.06, .58, .34)	α = .96 β = 1.23	Physionet	Data were differenced to provide beat intervals. 1/f noise suggested by both DFA and PSD. The AR model includes a mean of 964.6 ms.
**Psychology/ Neuroscience:**				
Relaxed Cognition (Resting EEG)	p = 7 (-.84, -.65, -.33, -.26, -.15, -.13, -.04)	α = .25 β = -1.18	Texas Data Repository	EEG Data were down-sampled to 8Hz (original was 256Hz) and differenced to provide EEG voltage change measures. The AR model includes a mean of -.28 microvolts. DFA and PSD suggest a hyper-corrective process, but not 1/f.

1/f properties were determined as described in the body of the paper (α refers to the DFA exponent; and β to the PSD); autoregressive models were constrained such that (p,d,q) were (p< = 10, 0,0) where p is the autoregressive order, d, the degree of differencing, and q the MA order (auto.arima from the R forecast library was used). The data used are from publicly available datasets, and are described in more detail in the Methods section.

Our argument in this paper is that rather than necessarily involving long-range correlations, 1/*f* features may sometimes be consequent simply of short range autocorrelations that involve multiple time-lags. In Table 3—together with the drumming data discussed in the body of this paper—we summarize the analyses we undertook on available non-musical time series datasets concerning unconstrained gait, relaxed heart beats, and relaxed cognition, each in the case of healthy human participants. These processes are chosen because of their diversity and oft-found 1/*f* characteristics. Table 3 therefore asks whether it is commonly the case that time series involving 1/*f* can be described in terms of autoregressive models of the multi-lag kind we have demonstrated above (where relatively low AR orders are required and can readily generate 1/*f* given appropriate ranges of AR coefficients). Indeed, the table suggests that a wide variety of 1/*f* processes can be interpreted as short-range autoregressive series with several orders, and hence our argument potentially has considerable generality beyond musical timing.

## Conclusions and discussion

To summarize, first we will revisit our opening questions. 1) ARIMA (short-range) simulations of the hi-hat series (with multiple time lags) did produce moderate-strong 1/*f* structures. 2) “Long-range” structures can result from a variety of short-range, ARIMA structures, and this is detectable by both PSD and DFA. This was even seen in the unusual case of large AR models with non-significant lags (that is, lags with zero-value coefficients in the model) between significant ones. We have also demonstrated some AR structures that reliably produce 1/*f*, namely those with positive coefficients at AR1. 3) The coexistence of low order AR and 1/*f* in a small but diverse set of human processes suggests that the short-range correlations deserve broader consideration as an explanation for 1/f processes, and specifically, those involving multiple lags.

DFA does seem to be a useful and generally reliable method of assessing fluctuation patterns in time series. However, one must consider the nature of the data to choose the proper parameters such as order of detrending [61], minimum and maximum window size, and regression range (which can greatly affect alpha). Particularly in musical contexts, the observations under consideration should be sampled regularly and unbroken series used; intentional irregularities in pulse such as different rhythmic values and expressive timing need to be treated with caution.

Many issues remain unresolved in relation to 1/f processes. As mentioned at the outset, the most stringent definitions of 1/f are often not fulfilled, and the PSD and DFA approaches both assume than an estimated linear gradient in the appropriate range is an adequate assessment, when it is not [1, 15]: genuine stringent 1/f processes are clearly a subset of those currently studied under the umbrella of 1/f. It has been investigated whether a reasonable basis for assessing stringent LRD might be to compare ARFIMA (fractionally differenced) models, with a nested AR1 model [15]. However even that work showed by simulation that a 1/f process generated by superposition of 3 AR1 systems was generally judged as LRD in that the ARFIMA model was stronger (as judged by the Aikake Information Criterion) than the AR1, yet the judgement was clearly misleading given the known origins.

In relation to both the stringent and the more loosely defined set of 1/f processes, the most important remaining issue for music and cognition is the nature of the processes which create the short-term temporal correlations that appear as 1/f. Our study does not directly address this, but we have pointed out that hierarchies of rhythmic structure contain the appropriate multiple time-scales. Thus as illustrated by the analysis of sequential hit intensities by the expert drummer, a 4/4 bar contains 4 beats each commonly divided into 4 semiquavers (16^th^ notes), and there is generally graded accentuation of semiquavers 1 and 9 > 3, 5, 7, 11, 13, 15 > the remainder. There are also correlations at the bar length. These involve motor and cognitive control patterns operating distinctively over these several different lags. Indeed, some evidence points to the most accentuated positions as being positions of peak attention on the part of a performer. Additionally, musical phrasing (operating commonly over several bars), and higher-level structures, such as 32 bar song pattern repetitions, provide other short to moderate time scales over which correlations contributing to an appearance of 1/f may be important. These considerations suggest several ways forward towards unravelling the origins of the 1/f property.

To conclude, we encourage rhythm and timing researchers, as well as those concerned with a broad range of other human activities within movement, physiology and neuroscience to consider interpretations of observed fractal and spectral structures other than LRD. The nature of time series with which they work (e.g. music) may be better explained by short-range influence. As such, researchers might consider using ARMA modeling to see if observed time series phenomena could possibly be attributed to repetitive activity with a small temporal range of influence. It is worth questioning if participants in a given experimental task might truly have “long memory” for the activity being measured, or if the activity could reasonably be influenced by the immediately preceding events or conditions. In many cases of human performance, the latter seems more likely.

Thinking beyond analytical methods, the occurrence and psychoacoustic impact of 1/*f* may be traced back to pre-musical physical activities such as vocalization [62]. Given these necessarily involved generation by SRD, they may have entrained preferential perception and cognition over evolutionary time scales. The selective value of this might be in facilitating localization and identification of different entities and physical processes in the environment. The utility of 1/*f* temporal processes in musical expression, like that of musical pitch relationships [49], may flow from these evolutionary considerations. Indeed computational creativity approaches to generating keyboard music from multi-lag SRD models have been developed [63]. Even electroacoustic music composers may be intuitively driven to include such features (in humanizing drum rhythms, and in spectral choices for example) and this forms an interesting path for future studies linking creativity and its effects to psychoacoustics and physical processes. Thus, 1/*f* structures may certainly be relevant to music composition and to many other human processes, but may often be best explained by processes that are short in time scale.

## Materials and methods

We used various time series analysis functions in R Studio to fit models and simulate data. These included auto.arima in the forecast library, various functions in the tseries library, dfa in the nonlinearTseries library, and arima.sim in the stats package. The R functions are mentioned as the models and simulations are described in the Results.

Our use of DFA required us to select two parameters: the window size range, and the regression range, the latter of which can greatly affect the alpha value. This is because the slope relating window size to fluctuation tends to flatten at larger window sizes, and can decrease alpha. All simulations were 1,024 observations, all window size ranges were 4 to *N*/2 (i.e. 512), and our regression range was 1–200. This range covered the linear region of the regression (i.e. before the slope flattens).

Much of our analysis was based on the inter-pulse time intervals and amplitudes from a hi-hat drum recording [45], which is included in the supporting information as S1 Dataset. However, for our initial ARIMA(X) models, the hi-hat data series required gap-filling due to missing pulses in the recording. While the original analysis of the hi-hat recording removed gaps in the pulse series and stitched together the fragments, we used an interpolation to fill missing parts of the recording. To do this we assumed an equal division of each interval into 16^th^ notes (i.e. the fastest rate of attack in the performance) and filled in missing pulses with that value. However, for the remaining simulations which were intended to reflect the original treatment of these data, we based our simulations on models that used the same data cleaning as the first paper that analyzed this drum performance [45].

We also used three non-music-related datasets from data repositories. Those were resting EEG, heart beat peak times, and walking gait time intervals. The resting EEG data are from Logan Trujillo 2017 in the Texas Data Repository (https://dataverse.tdl.org/dataset.xhtml?persistentId=doi:10.18738/T8/EG0LJI). Specifically we analyzed the Cz electrode, sampled at 256Hz from Study 4, Resting Subject S1. We down-sampled to 8Hz to bring the time intervals to roughly the same order of magnitude as those for the other processes we considered. The heart beat peak times were from Physionet [64], the PTB Diagnostic ECG Database, subject 104 control (https://physionet.org/physiobank/database/ptbdb/patient104/). Cumulative beat times were extracted using Physionet’s gqrs algorithm. The data were differenced to provide successive beat durations. The gait interval data came from subject 01 from data set ‘umwdb’, the Unconstrained and Metronomic Walking database of Physionet (https://physionet.org/physiobank/database/umwdb/si01.norm). The raw data represent spontaneous normal cumulative stride times, in seconds. The successive stride durations were obtained by differencing the data [65].

## Supporting information

S1 Table The various moving average (MA) filters represent influences of different coefficients and time lags (e.g. every 2, 4, and 6 observations; time lags in brackets) on the time series. (DOCX)

S1 Dataset The hi-hat drum recording [45] presented as 1) a cumulative time series of pulse intervals and 2) the amplitude of each drum hit.
(CSV)
