# Supplementary material for: Origins of 1/f noise in human music performance from short-range autocorrelations related to rhythmic structures
Source: PLoS One. 2019 May 6;14(5):e0216088. doi: 10.1371/journal.pone.0216088 (PMC6502337; doi:10.1371/journal.pone.0216088)
Supplement: Supplementary file 1 [file pone.0216088.s001.docx]

Table S1. Scaling exponents from simulated time series derived from white nois

Simulation Number	Formula	DFA α Mean	DFA α SD	PSD β Mean	PSD β SD
1	$WN+1\,MA(2)+1\,MA(4)+1\,MA(6)$	0.71	0.04	0.69	0.06
2	$WN+1\,MA(3)+1\,MA(6)+1\,MA(9)$	0.78	0.04	0.66	0.06
3	$WN+1\,MA(4)+1\,MA(8)+1\,MA(12$	0.82	0.04	0.63	0.06
4	$WN+1\,MA(4)+1\,MA(10)+1\,MA(1$	0.85	0.04	0.58	0.06
5	$WN+1\,MA(2)+1\,MA(4)+1\,MA(8)$	0.72	0.04	0.68	0.06
6	$WN+1\,MA(3)+1\,MA(9)+1\,MA(27$	0.78	0.04	0.58	0.06
7	$WN+1\,MA(4)+1\,MA(16)+1\,MA(6$	0.77	0.04	0.50	0.06
8	$WN+1\,MA(5)+1\,MA(25)+1\,MA(1$	0.76	0.04	0.44	0.06
9	$WN+1\,MA(2)+2\,MA(4)+3\,MA(6)$	0.78	0.04	0.92	0.06
10	$WN+1\,MA(3)+2\,MA(6)+3\,MA(9)$	0.87	0.04	0.89	0.06
11	$WN+1\,MA(4)+2\,MA(8)+3\,MA(12$	0.93	0.04	0.85	0.06
12	$WN+1\,MA(5)+2\,MA(10)+3\,MA(1$	0.97	0.04	0.79	0.06
13	$WN+1\,MA(2)+2\,MA(4)+3\,MA(8)$	0.81	0.04	0.90	0.06
14	$WN+1\,MA(3)+2\,MA(9)+3\,MA(27$	0.91	0.05	0.75	0.06
15	$WN+1\,MA(4)+2\,MA(16)+3\,MA(6$	0.88	0.05	0.63	0.06
16	$WN+1\,MA(5)+2\,MA(25)+3\,MA(1$	0.84	0.05	0.53	0.06
17	$WN+2\,MA(2)+4\,MA(4)+16\,MA(6$	0.84	0.04	1.26	0.06
18	$WN+2\,MA(3)+4\,MA(6)+16\,MA(9$	0.96	0.04	1.28	0.06
19	$WN+2\,MA(4)+4\,MA(8)+16\,MA(1$	1.04	0.04	1.28	0.06
20	$WN+2\,MA(5)+4\,MA(10)+16\,MA($	1.10	0.04	1.24	0.06
21	$WN+2\,MA(2)+4\,MA(4)+16\,MA(8$	0.90	0.04	1.26	0.06
22	$WN+2\,MA(3)+4\,MA(9)+16\,MA(2$	1.09	0.05	1.14	0.06
23	$WN+2\,MA(4)+4\,MA(16)+16\,MA($	1.07	0.06	0.99	0.06
24	$WN+2\,MA(5)+4\,MA(25)+16\,MA($	1.01	0.05	0.86	0.06
25	$WN+3\,MA(2)+9\,MA(4)+27\,MA(6$	0.84	0.04	1.35	0.06
26	$WN+3\,MA(3)+9\,MA(6)+27\,MA(9$	0.97	0.04	1.40	0.06
27	$WN+3\,MA(4)+9\,MA(8)+27\,MA(1$	1.05	0.04	1.42	0.06
28	$WN+3\,MA(5)+9\,MA(10)+27\,MA($	1.12	0.04	1.40	0.06
29	$WN+3\,MA(2)+9\,MA(4)+27\,MA(8$	0.90	0.04	1.35	0.06
30	$WN+3\,MA(3)+9\,MA(9)+27\,MA(2$	1.12	0.05	1.31	0.06
31	$WN+3\,MA(4)+9\,MA(16)+27\,MA($	1.13	0.05	1.20	0.06
32	$WN+3\,MA(5)+9\,MA(25)+27\,MA($	1.10	0.05	1.08	0.06
33	$WN+27\,MA(2)+9\,MA(4)+3\,MA(6$	0.67	0.04	1.10	0.06
34	$WN+27\,MA(3)+9\,MA(6)+3\,MA(9$	0.75	0.04	1.24	0.06
35	$WN+27\,MA(4)+9\,MA(8)+3\,MA(1$	0.83	0.04	1.36	0.06
36	$WN+27\,MA(5)+9\,MA(10)+3\,MA($	0.89	0.04	1.39	0.06
37	$WN+27\,MA(2)+9\,MA(4)+3\,MA(8$	0.67	0.04	1.09	0.06
38	$WN+27\,MA(3)+9\,MA(9)+3\,MA(2$	0.76	0.04	1.24	0.06
